# Distal Humeral Shaft Fracture with Simultaneous Ipsilateral Supracondylar Humeral Fracture in a Child: A Case Report

**DOI:** 10.5704/MOJ.2107.026

**Published:** 2021-07

**Authors:** JA Talal, NA Abdulmuhsen, MK Rakan

**Affiliations:** Department of Orthopaedic, King Fahad General Hospital, Al-Madinah, Saudi Arabia

**Keywords:** antegrade, children, elastic stable intramedullary nails, supracondylar humeral fracture, internal fixation

## Abstract

In children, simultaneous ipsilateral fracture of the distal shaft humerus and supracondylar humerus is rare. To the best of our knowledge, there are no available data on supracondylar humeral fracture (SCHF) combined with ipsilateral distal humeral shaft fracture. This report aimed to discuss a novel case with an appropriate management scheme. We present a case of a 5-year-old boy with ipsilateral distal shaft humerus fracture and SCHF. He underwent closed reduction and antegrade elastic stable intramedullary nail (ESIN) fixation with good clinical outcome. These injuries affect a child’s bony growth, and careful treatment is warranted. Upper extremity fractures could present in different combinations. The ESIN procedure with antegrade technique facilitated reduction and fixation of the fracture by prevention of iatrogenic ulnar nerve injury or pin-tract infection. Moreover, it allowed performance of early range of motion exercises.

## Introduction

Simultaneous ipsilateral fracture of the distal humeral shaft and supracondylar humerus is rare, although fractures around the elbow are frequent among the paediatric population. Approximately 57% are supracondylar, and 2.5% combined elbow fractures^1^. Dealing with such a rare fracture would help expand knowledge of how to manage the fracture and establish a suitable treatment scheme. Keeping this in mind, these injuries affect a child’s bony growth, and adequate treatment is crucial.

This report discusses our experience in the treatment of this rare fracture; a different method has been explained in the treatment of supracondylar fracture^2^, but there is insufficient available data on ipsilateral distal humerus with a supracondylar humeral fracture. Our treatment aimed to restore the alignment of distal humerus fracture, achieve an acceptable reduction of supracondylar fracture with less intra-operative risk, and begin performance of early range of motion exercises after operation. We chose elastic stable intramedullary nails (ESINs) with an antegrade technique, which was appropriate with our management goals.

## Case Report

**Fig. 1:**
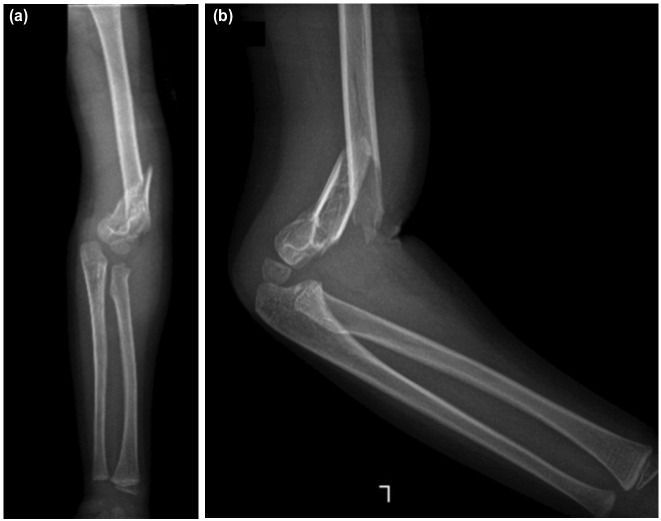
(a) Initial post-traumatic anteroposterior and (b) lateral radiographs of the left elbow showing ipsilateral fracture of the distal shaft humerus and supracondylar with complete displacement.

A 5-year-old boy presented to our emergency department following an unwitnessed fall from the kitchen countertop (approximately one meter in height). The patient complained of left elbow pain, deformity, and swelling. Physical examination showed normal vital signs. The deformity was noted at the distal part of the left arm with severe left elbow swelling. Distal neurovascular assessments were unremarkable. Left upper limb radiography showed extension-type left supracondylar humerus Gartland type 2 fracture with distal humeral shaft fracture (Fig. 1). The patient was admitted and scheduled to undergo urgent closed reduction and internal fixation. The procedure was conducted under general anaesthesia with endotracheal intubation.

The patient was placed in the supine position with radiolucent arm support. Under image guidance, preliminary realignment was achieved with simple inline traction. A 2cm skin incision was made following the line of the deltopectoral groove on the anterior aspect of the left arm. Soft tissue was dissected in layers until the anterolateral aspect of the humerus was reached. The periosteum was incised, and 2 Hoffman retractors were placed subperiosteally. The entry point was made using a 4.5mm drill bit; then, an awl was introduced to widen the hole. Two 2.5mm elastic nails were contoured and prepared. The 2 ESINs were inserted and advanced until it passed the proximal fracture line; through this manoeuvre, closed reduction for the distal humerus fracture was achieved.

**Fig. 2:**
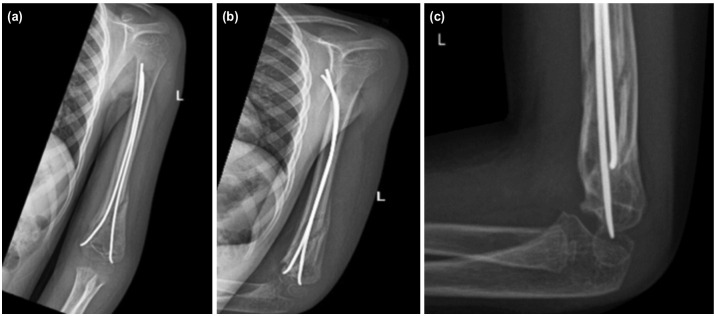
(a) Anteroposterior, (b) oblique and (c) lateral radiographs of the left arm and elbow at three months showing fractures healed in good position.

**Fig. 3:**
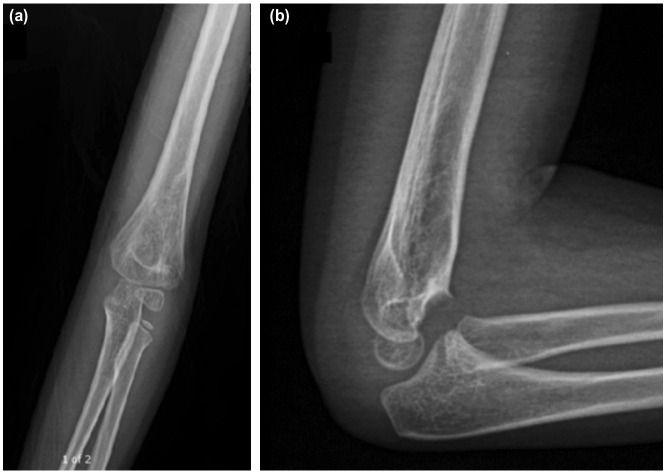
Final follow-up radiographs at 10 months. (a) Anteroposterior and (b) lateral of the left elbow demonstrating complete fracture healing.

Then, the SCHF was reduced by the usual manoeuvre, and the ESINs were advanced deeply until the humeral condyles. Reduction and fixation were assessed under fluoroscopy. The above-elbow back slab was applied. Post-operatively, the patient was transferred to the orthopaedic ward for pain control, compartment, and neurovascular status monitoring. The patient was discharged on the second post-operative day in good condition with normal neurovascular examinations. On follow-up visit at two weeks, the wound was completely healed. Stitches and back slab were removed, and elbow range of motion was allowed as tolerated. At six weeks postoperatively, examinations revealed no elbow deformity. Left upper limb radiography showed good alignment with adequate callus formation (Fig. 2). After three months, the patient was booked for removal of ESIN under general anaesthesia; intra-operatively, the arc elbow motion was 0° to 100°. At 10 months of follow-up, the patient was asymptomatic, and the elbow arc of flexion–extension was 0° to 130° (Fig. 3).

Written informed consent to publish this case report and its accompanying images was obtained from the patient’s father.

## Discussion

Concomitant ipsilateral fractures of the distal shaft humerus and supracondylar humerus in the paediatric population are considered rare; no other cases with the same injury have been reported in English literature, although elbow fracture is deemed to be common in children^1^. Our patient had Gartland type 2 supracondylar fracture with ipsilateral distal humeral shaft fracture. The distal humerus metaphyseal–diaphyseal junction has a thinner periosteum than the supracondylar humerus, making it less stable than that in simple supracondylar fracture^3^. Additionally, diaphyseal fracture requires more time for healing than metaphyseal fracture; hence, stable and prolonged immobilisation is required for this fracture type^3^. In our case, because of the fracture geometry and combination of 2 fractures in a small area, we chose to perform closed reduction and antegrade intramedullary fixation for both fractures using ESIN. This technique helped us achieve reduction by advancing the ESIN until it passed the proximal fracture line converting the fracture from 3 to 2 parts. SCHF was reduced in the coronal and sagittal planes with respect to malrotation correction; then, the ESINs were advanced into the bilateral condyles. However, we paid more attention to coronal plane reduction, and some extension in sagittal plane was accepted, as the displacement of SCHF in sagittal plane has a greater capacity of remodelling with a good functional outcome compared to coronal plane.

Furthermore, by ESIN fixation in our case, we mitigate the iatrogenic ulnar nerve injury risk compared to crossed Kirschner wire fixation, with rates reaching up to 3% to 4%. In Kirschner wire fixation, there might be difficulties in pin insertion, as epicondyles are not yet ossified. However, ESIN fixation allows performance of early range of motion at the elbow and results in less immobilisation time and risk of pin-tract infection^4^. Moreover, no varus deformity cases were reported following ESIN fixation, which also was concurred by other authors^5^. In contrast, longer time of elastic nail removal (mean, 55 days)^2^ and the need for further surgery for ESIN removal are considered disadvantages for this technique compared to the Kirschner wire fixation, which can be easily removed in the orthopaedic follow-up clinic.

In conclusion, ipsilateral fractures of the distal humeral shaft and supracondylar humerus are considered a rare injury. ESIN fixation is a reliable method and helpful tool in achieving closed reduction. Compared with Kirschner wire fixation, ESIN fixation reduces the risk of iatrogenic ulnar nerve injury and offers more stable fixation; hence, prolonged time for splinting is not required.
